# Disruption of *TWIST1* translation by 5′ UTR variants in Saethre‐Chotzen syndrome

**DOI:** 10.1002/humu.23598

**Published:** 2018-08-07

**Authors:** Yan Zhou, Nils Koelling, Aimée L. Fenwick, Simon J. McGowan, Eduardo Calpena, Steven A. Wall, Sarah F. Smithson, Andrew O.M. Wilkie, Stephen R.F. Twigg

**Affiliations:** ^1^ Clinical Genetics Group, MRC Weatherall Institute of Molecular Medicine University of Oxford Oxford UK; ^2^ Analysis, Visualisation and Informatics Group, MRC WIMM Centre for Computational Biology MRC Weatherall Institute of Molecular Medicine University of Oxford Oxford UK; ^3^ Craniofacial Unit, Department of Plastic and Reconstructive Surgery, Oxford University Hospitals NHS Foundation Trust, John Radcliffe Hospital University of Oxford Oxford UK; ^4^ Department of Clinical Genetics, St Michaels Hospital & School of Clinical Sciences University of Bristol Bristol UK

**Keywords:** haploinsufficiency, Saethre‐Chotzen syndrome (SCS), TWIST1, upstream AUG (uAUG), upstream open reading frame (uORF)

## Abstract

Saethre‐Chotzen syndrome (SCS), one of the most common forms of syndromic craniosynostosis (premature fusion of the cranial sutures), results from haploinsufficiency of *TWIST1*, caused by deletions of the entire gene or loss‐of‐function variants within the coding region. To determine whether non‐coding variants also contribute to SCS, we screened 14 genetically undiagnosed SCS patients using targeted capture sequencing, and identified novel single nucleotide variants (SNVs) in the 5′ untranslated region (UTR) of *TWIST1* in two unrelated SCS cases. We show experimentally that these variants, which create translation start sites in the *TWIST1* leader sequence, reduce translation from the main open reading frame (mORF). This is the first demonstration that non‐coding SNVs of *TWIST1* can cause SCS, and highlights the importance of screening the 5′ UTR in clinically diagnosed SCS patients without a coding mutation. Similar 5′ UTR variants, particularly of haploinsufficient genes, may represent an under‐ascertained cause of monogenic disease.

Craniosynostosis, a malformation of skull development caused by premature fusion of one or more of the cranial sutures, affects around 1 in 2100 children (Lajeunie, Le Merrer, Bonaïti‐Pellie, Marchac, & Renier, 1995). A genetic cause accounts for ∼25% of craniosynostosis cases, most frequently due to coding mutations in *FGFR2*, *FGFR3*, and *TWIST1* (Wilkie, Johnson, & Wall, 2017). Heterozygous mutations of *TWIST1* (MIM# 601622) result in Saethre‐Chotzen syndrome (SCS; MIM# 101400) and typical features include coronal craniosynostosis, hypertelorism, ptosis, low frontal hairline, blocked tear ducts, and small dysmorphic ears (El Ghouzzi et al., 1997; Howard, et al., 1997). *TWIST1* encodes a basic helix–loop–helix transcription factor that regulates a variety of processes, including calvarial development, where it has important roles in boundary formation at the coronal suture (Merrill et al., 2006) and in inhibiting premature osteogenesis in sutural mesenchyme (Bialek et al., 2004; Yen, Ting, & Maxson, 2010). TWIST1 binds DNA as a homo‐ or heterodimer and the key basic helix–loop–helix partner in coronal suture formation and integrity is TCF12 (Sharma et al., 2013). Heterozygous loss‐of‐function point mutations within the *TWIST1* coding region and monoallellic whole‐gene deletions have been reported in patients with SCS, consistent with haploinsufficiency of *TWIST1* as the underlying causative mechanism (El Ghouzzi et al., 1997; Howard, et al., 1997; Johnson et al., 1998). As reduced expression of *TWIST1* could also be caused by mutation of non‐coding regulatory elements, we set out to screen the entire gene in SCS cases who were negative for known causes of craniosynostosis.

As part of a wider study, we designed a resequencing capture panel to the *TWIST1* gene and flanking regions (2.4 Mb design with boundaries selected using human to mouse synteny; chr7:17346143‐19695462, GRCh38) and used this in the analysis of 14 SCS cases in whom no mutation of *TWIST1* or other craniosynostosis‐associated genes had been identified (genetic screening was documented in all cases for *TWIST1*, and in the majority of cases for *TCF12*, *FGFR2* exons IIIa and IIIc, and FGFR3 exon7 (Wilkie et al., 2017)). Ethical review board approval [Oxfordshire Research Ethics Committee B (reference C02.143) and Riverside Research Ethics Committee (reference 09/H0706/20)] and informed, written consent from the families was received for the study. Genomic DNA was extracted from venous blood samples, sonicated and ligated to indexed Illumina sequencing adapters. Amplified libraries were pooled for capture with a biotinylated probe mixture (SeqCap EZ Choice Library system, Roche‐Nimblegen). Genomic DNA enriched for the targeted regions was subsequently sequenced on either Illumina HiSeq 2500 or NextSeq 500 platforms. Read pairs were trimmed to remove sequencing adapters and low‐quality bases using Trimmomatic (v0.32, parameter SLIDINGWINDOW:4:20) (Bolger, Lohse, & Usadel, 2014). Trimmed read pairs were aligned to human reference genome hg19 using BWA (v0.7.12) in paired‐end mode with default parameters (Li & Durbin, 2009). Target coverage was calculated using BEDtools v0.25.0 (Quinlan & Hall, 2010) and processed using amplimap (v0.2.9, https://github.com/koelling/amplimap). An average depth of >100× was achieved (Supp. Table S1). Variants were called separately in each sample using Platypus (v0.8.1) (Rimmer et al., 2014). Variant calls were then concatenated, merged, and normalized using BCFtools (v1.5, https://github.com/samtools/bcftools) and annotated using Annovar (Wang, Li, & Hakonarson, 2010).

Here, we report on our analysis of the *TWIST1* genomic sequence. We searched (June 2017) for variants that were not listed in public databases of variation, including the 1000 Genomes Project (https://www.internationalgenome.org) and gnomAD (https://gnomad.broadinstitute.org), and this identified three variants within the entire *TWIST1* sequence, all within the 5′ UTR, in 2 of the 14 SCS probands (Supp. Figure S1A; variants have been deposited in the Leiden Open Variation Database: https://www.lovd.nl/TWIST1). In Family 1, two heterozygous variants were present in *cis* in the proband III‐3 (c.‐281G > T and c.‐263C > A (NM_000474.3); GRCh38: chr7:19117602C > A and 19117584G > T, respectively). This child had a clinically affected mother and brother (II‐2 and III‐1, respectively; Figure 1A) and dideoxy‐sequencing of the *TWIST1* 5′ UTR (primers and amplification conditions are shown in Supp. Table S2) confirmed the presence of both variants in all three affected individuals (Figure 1B). The proband presented with right unicoronal synostosis, hypertelorism, and facial asymmetry (Figure 1C). His mother and brother had mild facial features suggestive of SCS, together with limb anomalies (wide sandal gap in III‐1 and webbing between the 4th and 5th toes in both II‐2 and III‐1; Figure 1C).

**Figure 1:**
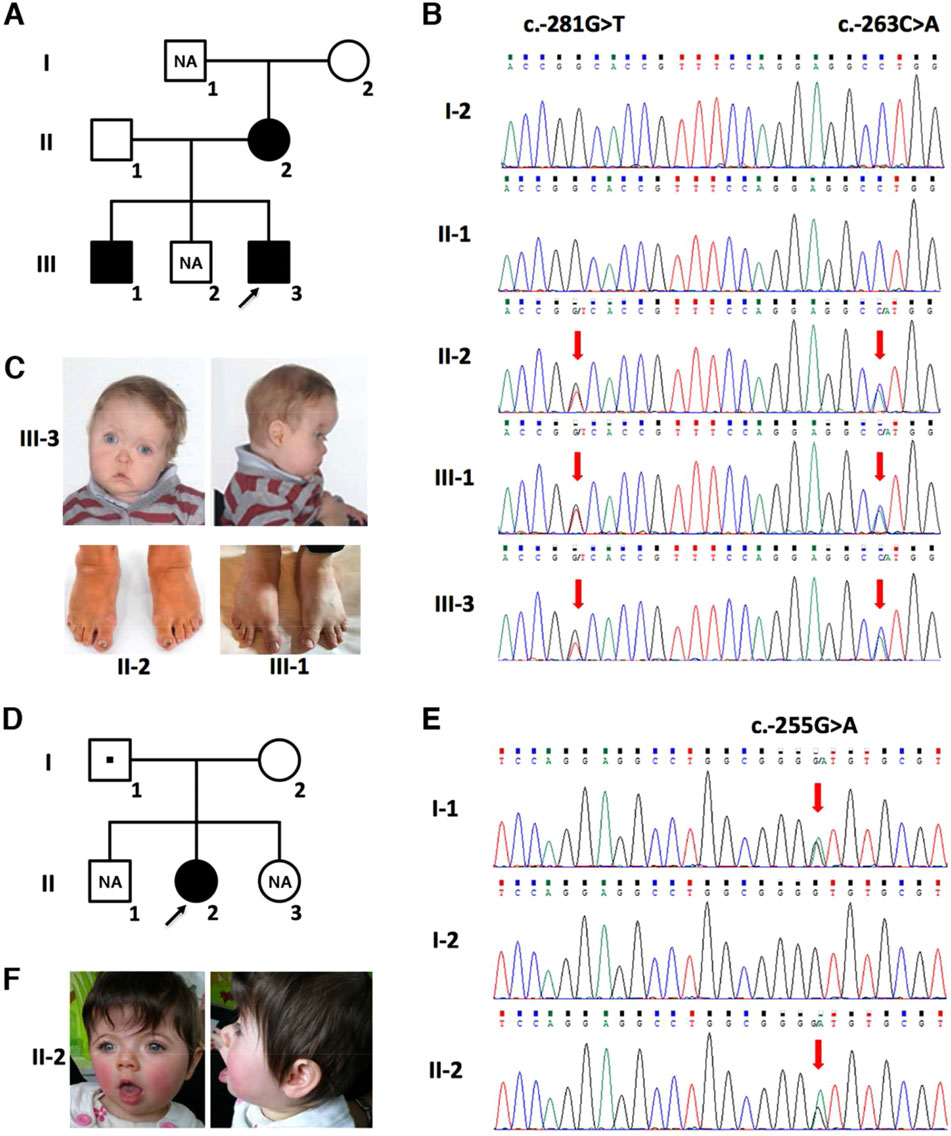
Identification of *TWIST1* 5′ UTR variants in SCS. A: Pedigree of Family 1. Affected individuals are indicated by filled squares or circles. DNA was not available (NA) from I‐1 and III‐2. B: Validation of *TWIST1* 5′ UTR variants by dideoxy‐sequencing of genomic DNA isolated from peripheral blood or saliva in Family 1: The heterozygous variants c.‐281G > T and c.‐263C > A (ATG) are indicated by red arrows. C: Clinical photographs of III‐3 (top, preoperative aged 10 months) and II‐2 and III‐1 (bottom). Note facial asymmetry due to right coronal synostosis in III‐3, webbing of 4th and 5th toes in II‐2 and III‐1, and wide sandal gap in III‐1. D: Family 2 pedigree. The variant identified in II‐2 was inherited from the clinically unaffected father I‐1 (square with central dot). E: Dideoxy‐sequence traces from the *TWIST1* 5′ UTR with the c.‐255G > A variant indicated by red arrows. F: Preoperative facial appearance of the Family 2 proband II‐2 aged 9 months. Note: hypertelorism and brachycephaly due to bicoronal synostosis

In Family 2 (Figure 1D), a single *TWIST1* variant c.‐255G > A (GRCh38: chr7:19117576C > T) was identified in the proband, II‐2 (Supp. Figure S1B). Dideoxy‐sequencing showed that this variant was inherited from the apparently unaffected father (I‐1; Figure 1E). Mosaicism of the variant in I‐1 was excluded in DNA from both peripheral blood and saliva by deep sequencing (data not shown). II‐2 had bicoronal synostosis with brachycephaly, mild hypertelorism, and facial appearance consistent with SCS (Figure 1F). She had clinodactyly of the 5th fingers and bilateral single palmar creases. Although no other family members had craniosynostosis, her father had bilateral single palmar creases.

Inspection of the sequence context around the three 5′ UTR variants revealed that c.‐263C > A (Family 1) and c.‐255G > A (Family 2) create upstream AUG (uAUG) translation initiation codons 5′ of the *TWIST1* main ORF (mORF; Figure 2A); importantly, no such sequences are present in the wild‐type (WT) *TWIST1* 5′ UTR, either in humans or in all other vertebrate species that we were able to analyse (Supp. Figure S2). The sequence contexts at these positions both provide good matches with the Kozak consensus (Kozak, 1986) for translation initiation, and analysis using the prediction tools DNA functional site miner (DNAFSMiner; https://dnafsminer.bic.nus.edu.sg/), NetStart (https://www.cbs.dtu.dk/services/NetStart/), and ATGpr (https://atgpr.dbcls.jp/) suggested that both uAUGs could potentially compete with the endogenous *TWIST1* start AUG (sAUG) as translation initiation sequences (Figure 2B). A purine at ‐3 from the AUG is the most functionally important residue (Kozak, 1986) and all three possible start sites harbor a guanine. A guanine residue at the +4 position is also preferred and by this criterion, the ‐263 uAUG has a stronger context than the sAUG. The c.‐263C > A variant generates an upstream open reading frame (uORF) of 68 codons that is out‐of‐frame with the main *TWIST1* coding ORF, and ends at a highly conserved stop codon (Supp. Figure S2), 59 bp upstream of the sAUG (Figure 2A). In contrast, the c.‐255G > A variant, located eight nucleotides downstream of c.‐263C > A, generates an uAUG in‐frame with the mORF, that if translated would add 85 amino acids to the TWIST1 protein. No mechanism was identified by which the c.‐281G > T variant might be pathogenic.

**Figure 2:**
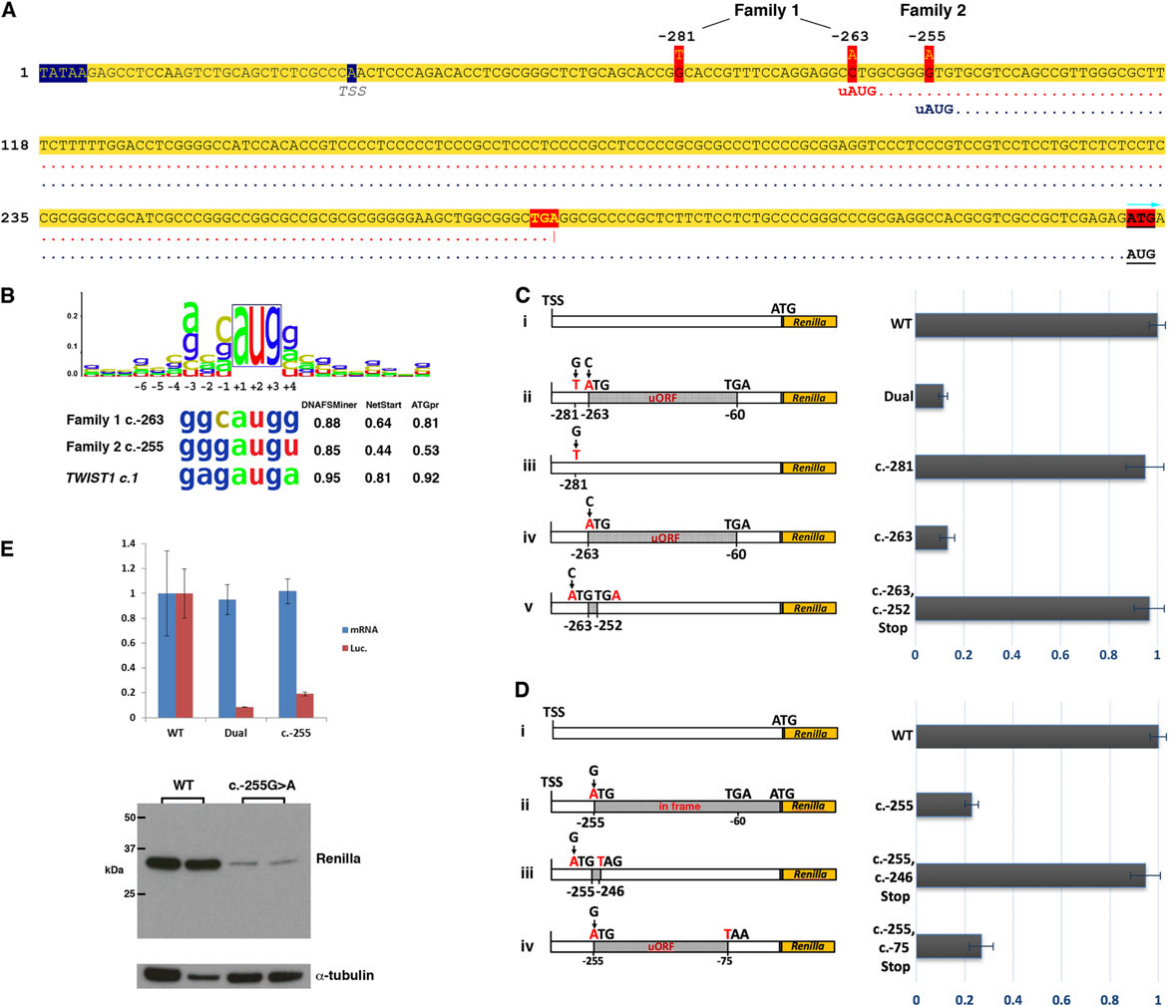
*TWIST1* 5′ UTR variants and effect on translation. A: Genomic sequence showing the locations of the variants identified in Families 1 and 2 within the 5′ UTR of *TWIST1 (*NM_000474.3). The TATAA box and transcription start site (TSS) are denoted by blue shading. The reading frames from the uAUGs at ‐263 (Family 1) and ‐255 (Family 2) are indicated by red and blue dotted lines, respectively. Note that the ORF from c.‐263 terminates at a stop codon (TGA; yellow text with red shading) that is 62 bp upstream of the reference start codon of *TWIST1* (denoted by red highlighting and turquoise arrow). The uAUG in Family 2 is in‐frame with the *TWIST1* start codon. B: Kozak consensus sequence (Kozak, 1986) aligned to the uAUGs of Families 1 and 2, and to the *TWIST1* start codon (sAUG). The relative strengths of these possible translation initiation sequences were assessed by three online tools, DNA functional site miner (DNAFSMiner), NetStart, and ATGpr with scores shown on the right. C: Luciferase analysis to determine the effect of the Family 1, 5′ UTR variants on translation. Luciferase reporter DNA constructs are shown on the left and normalized luciferase activity generated from each is shown on the right. (i) WT construct. (ii) The Dual construct contains both c.‐263 and c.‐281 variants, while the c.‐281 (iii) and c.‐263 (iv) constructs contain each variant in isolation. The c.‐263C > A variant is in‐frame with a TGA stop codon at c.‐62_‐60 generating a large uORF of 204 bp (grey shading; 68 codons). (v) The c.‐263, c.‐252Stop construct incorporates a new stop codon at ‐254_‐252, shortening the uORF to four codons and extending the distance from the uORF to the mORF from 59 bp to 251 bp. D: Luciferase analysis of the Family 2 variant c.‐255G > A. (i) WT construct. (ii) The ATG created by c.‐255 is in‐frame with the luciferase ORF adding a further 85 codons. (iii) The c.‐255, c.‐246Stop construct incorporates a new stop codon at ‐246_‐244 to create a short four codon uORF, while the c‐255, c.‐75Stop construct (iv) contains a longer uORF of 61 codons. Plots are shown as mean±standard error based on three separate experiments carried out in triplicate. E: The top panel shows RT‐qPCR (blue) and dual luciferase reporter (red) assays in HEK 293T cells comparing *Renilla* luciferase expression and activity using WT, Dual, and c.‐255 constructs (plots are shown as mean±SD). The *y*‐axis shows relative expression or activity of the *Renilla* reporter gene (normalized against firefly and to WT). mRNA levels and luciferase activity are indicated in blue and red, respectively. Bottom panel: western blot analysis of transfected HEK293 cell lysates showing expression of *Renilla* luciferase produced from WT (lanes 1 and 2) and c.‐255 constructs (lanes 3 and 4), from separate experiments. The N‐terminal extension produced by translation from c.‐255 uAUG is predicted to increase the molecular weight of *Renilla* by ∼9 kDa, but a larger product was not detected. Anti‐*Renilla* luciferase antibody (Abcam ab185925) and α‐tubulin (Santa Cruz, sc‐32293) at 1/1000 dilutions were used against 10 μg of protein lysate (BCA protein assay kit, Thermo)

To test whether any of the three 5′ UTR variants might be associated with down‐regulation of TWIST1 protein output, we carried out functional assays using a dual luciferase reporter transfected into HEK293T cells, as previously described (Calvo, Pagliarini, & Mootha, 2009; Twigg et al., 2013). The WT sequence of the full‐length *TWIST1* 5′ UTR was amplified and cloned into the psiCHECK‐2 dual‐luciferase reporter (Calvo et al., 2009), so that *Renilla* luciferase translation initiated at the sAUG of *TWIST1*. This construct was further modified by site‐directed mutagenesis (New England Biolabs) to introduce specific variants into the 5′ UTR sequence, including the individual variants carried by the two SCS probands (Supp. Table S2). All constructs were verified by dideoxy‐sequencing, and fluorimetric assays were performed to obtain the relative expression of *Renilla* luciferase to the internal Firefly luciferase control. First we assessed whether, individually or together, the c.‐281G > A and c.‐263C > A variants identified in Family 1 had an impact on translation. Constructs containing both variants, or c.‐263C > A alone, showed >80% reduction in relative *Renilla* activity compared to WT (88.51% ± 3.06% and 86.81% ± 5.26%, respectively), whereas there was no significant reduction observed with the c.‐281G > A variant alone (Figure 2C, i–v). This suggests that c.‐263C > A is the causal variant in Family 1 and supports the hypothesis that this variant negatively influences translation of the WT protein. To investigate this further, we assessed the impact of shortening the ‐263C > A uORF from 68 to 4 codons by introducing an earlier stop codon at c.‐252T > A, and found that the relative *Renilla* activity returned to WT levels (Figure 2C, v). This implies that both the length of the ‐263C > A uORF and the distance between its stop codon and the sAUG are important for the repressive effect on translation.

Reporter protein output from the construct containing the Family 2 c.‐255G > A variant was decreased by over 75% (77.19% ± 4.74%) compared to WT (Figure 2D, i–ii). As the ‐255 uAUG is in‐frame and has a slightly weaker Kozak consensus that the sAUG, our expectation was that two *Renilla* proteins differing by an 85 amino acid N‐terminal extension (∼9 kDa) would be produced. To investigate the relative reduction in *Renilla* luciferase activity further, we analyzed both the RNA and protein produced in the assay. We found no difference in the amount of RNA produced by the c.‐255 and WT constructs in a reverse transcription quantitative PCR (RT‐qPCR) analysis (normalized against firefly expression; for primers and methods, see Supp Table S2) of transfected HEK293 cells (Figure 2E). We then looked for expression of the larger protein by western blot analysis of reporter assay lysates using an antibody against Renilla (Abcam ab185925). This showed that the presence of the c.‐255 uAUG led to a dramatic reduction in Renilla expression, and that there was no evidence of a larger fusion protein (Figure 2E). *Renilla* expression was completely restored when a stop codon was introduced at c.‐246, suggesting that in the context of a small uORF (three codons), the uAUG does not substantially impact on translation from the sAUG. Finally, we confirmed that the ‐255 uAUG functions as a translation start site by using a construct with a uORF of similar size to that identified in Family 1 (Figure 2D, iv). This analysis showed a similar knock‐down effect on *Renilla* expression (73.16% ± 8.64%), supporting the fact that the c.‐255 uAUG is recognized and engaged by the translational machinery. Taken together, the luciferase data suggest that the c.‐255G > A variant could lead to suppressed translation from the sAUG, or preferential production of the N‐terminally extended protein which is highly unstable.

Regulatory elements within the 5′ UTR of mature mRNAs are important contributors to the post‐transcriptional control of gene expression and include uAUGs, uORFs, and internal ribosome entry sites (Mignone & Pesole, 2016). Translation of the majority of eukaryotic mRNAs is by the scanning mechanism, whereby the 43S preinitiation complex first binds to the 5′ cap, then scans along the leader sequence for the first AUG codon present in a suitable context. Secondary structure and elements such as uAUGs and uORFs can affect ribosome scanning efficiency and thus modulate the level of translation of the main coded protein, and both uAUGs and uORFs are found at a lower than expected frequency in 5′ UTRs (Iacono, Mignone, & Pesole, 2005). Approximately 50% of mammalian 5′ UTRs contain uORFs that generally act as repressive regulators of gene activity (Calvo et al., 2009; Johnstone, Bazzini, & Giraldez, 2016; Ye et al., 2015), with control of translation mediated through several different mechanisms (Cabrera‐Quio, Herberg, & Pauli, 2016; Wethmar, 2014). The number of diseases known to be caused by mutations that introduce or disrupt uORFs is increasing (Barbosa, Onofre, & Romao, 2014; Calvo et al., 2009; Chatterjee, Rao, & Pal, 2017) and, in this work, we show that a uORF‐generating variant (c.‐263C > A) in the 5′ UTR of *TWIST1* likely leads to SCS. Although there are >50 different SNVs within the TWIST1 5′ UTR catalogued in the gnomAD database, none creates an uAUG (Supp. Figure S3A), and *TWIST1* is unusual in having a relatively long 5′ UTR without an uAUG (Supp. Figure S3B). As implied by the *in vitro* analysis, translation of the ‐263 uORF within the *TWIST1* mRNA leader sequence is likely to lead to a reduction in mORF expression, resulting in the same phenotypic outcome as caused by deletions or loss‐of‐function mutations that affect the coding sequence. The reduction in expression of the mORF was not complete (88.52%) suggesting that either skipping (leaky scanning) of the mutant AUG could occur or that following translation of the uORF there is reinitiation of translation at the mORF. However, the complete penetrance (albeit with variable expressivity) in the three individuals heterozygous for the c.‐263C > A variant indicates that loss of TWIST1 activity was consistently below the threshold required for normal development.

Interpretation of the c.‐255G > A variant in Family 2 is more challenging, as the variant introduces an uAUG that is in‐frame with the main *TWIST1* coding sequence, and there was apparent incomplete penetrance of the SCS phenotype in the father I‐1. That in‐frame uAUGs can affect translation from the mORF is supported by the observation that such codons are suppressed in the 5′ UTRs of mammalian genes, strikingly even more so than uORFs or out‐of‐frame uAUGs (Iacono et al., 2005). Translation start site choice is influenced by distance from the cap, sequence context, secondary structure, and the availability of eukaryotic initiation factors (reviewed in Brar, 2016; Hinnebusch, Ivanov, & Sonenberg, 2016). If an uAUG is recognized by the preinitiation complex then this might act as a soak for ribosomes and moreover, translation of the mORF cannot occur through reinitiation but only through either leaky scanning, which will be influenced by the strength of the Kozak consensus, or perhaps through ribosome shunting, where parts of the 5′ UTR are physically bypassed. Our results show that although the c.‐255 uAUG sequence context is marginally weaker than that of the sAUG, it is recognized by the ribosomal machinery as translation of the mORF is reduced when the uAUG is in‐frame with the main coding sequence or a distant upstream termination codon. Translation resulting in N‐terminal extension because of an in‐frame uAUG (or “near‐cognate” translation start sites with a single base substitution of AUG) has been demonstrated by ribosomal profiling (Fields et al., 2015; Fritsch et al., 2012; Ingolia, Lareau, & Weissman, 2011). In a normal physiological setting this process may regulate translation of the primary ORF (Karagyozov et al., 2008; Song et al., 2010) as well as production of different isoforms (Calkhoven, Muller, & Leutz, 2000) and their subcellular localization (Touriol et al., 2003). However, a non‐physiological N‐terminal addition to a protein can have detrimental effects on structure, stability, or targeting. In relation to the *TWIST1* uAUG found in Family 2, factors such as AUG choice, stability, and function of an extended protein if produced, as well as expression levels from the WT allele, will in combination determine whether there is sufficient functional TWIST1 protein for development. This balance may be close to the *TWIST1* dosage threshold for normal development, providing a possible explanation for phenotypic variation found in the two mutation‐positive individuals in Family 2.

In summary, we have identified the first non‐coding point mutations in SCS, and demonstrate that they cause a reduction in TWIST1 expression at the level of translation. It is likely that similar variants are present in other dosage‐sensitive genes and represent an under‐ascertained pool of causal mutations within 5′ UTRs. Such regions are often excluded in diagnostic screening, or poorly covered because of GC‐richness, but with the increased use of, and improvement in, whole genome sequencing, more potentially pathological non‐coding variants will be identified and require clinical interpretation. In craniosynostosis, pathological variants have been identified in the 5′ UTRs of *EFNB1* (Romanelli Tavares et al., 2018; Twigg et al., 2013) and *SMAD6* (E.C., unpublished data), highlighting the importance of screening these sequences in patients with a clear diagnosis and where a coding mutation or deletion cannot be identified.

## CONFLICT OF INTEREST

The authors have no conflicts of interest to declare.

## Supporting information

Supplementary Figure S1. *TWIST1* 5′ UTR variants
Supplementary Figure S2. Alignment of vertebrate *TWIST1* 5′ UTR sequences
Supplementary Figure S3. *TWIST1* 5′ UTR‐SNVs and uATGs
Supplementary Table S1. Resequencing coverage statistics
Supplementary Table S2. Primers and amplification conditions
