# Supplementary material for: Disruption of TWIST1 translation by 5′ UTR variants in Saethre‐Chotzen syndrome
Source: Hum Mutat. 2018 Aug 7;39(10):1360–5. doi: 10.1002/humu.23598 (PMC6175480; doi:10.1002/humu.23598)
Supplement: Supplementary file 1 — Supplementary Figure S1. TWIST1 5′ UTR variants Supplementary Figure S2. Alignment of vertebrate TWIST1 5′ UTR sequences Supplementary Figure S3. TWIST1 5′ UTR‐SNVs and uATGs Supplementary Table S1. Resequencing coverage statistics Supplementary Table S2. Primers and amplification conditions [file HUMU-39-1360-s001.pdf]

Supplementary Figure S1. *TWIST1* 5′ UTR variants

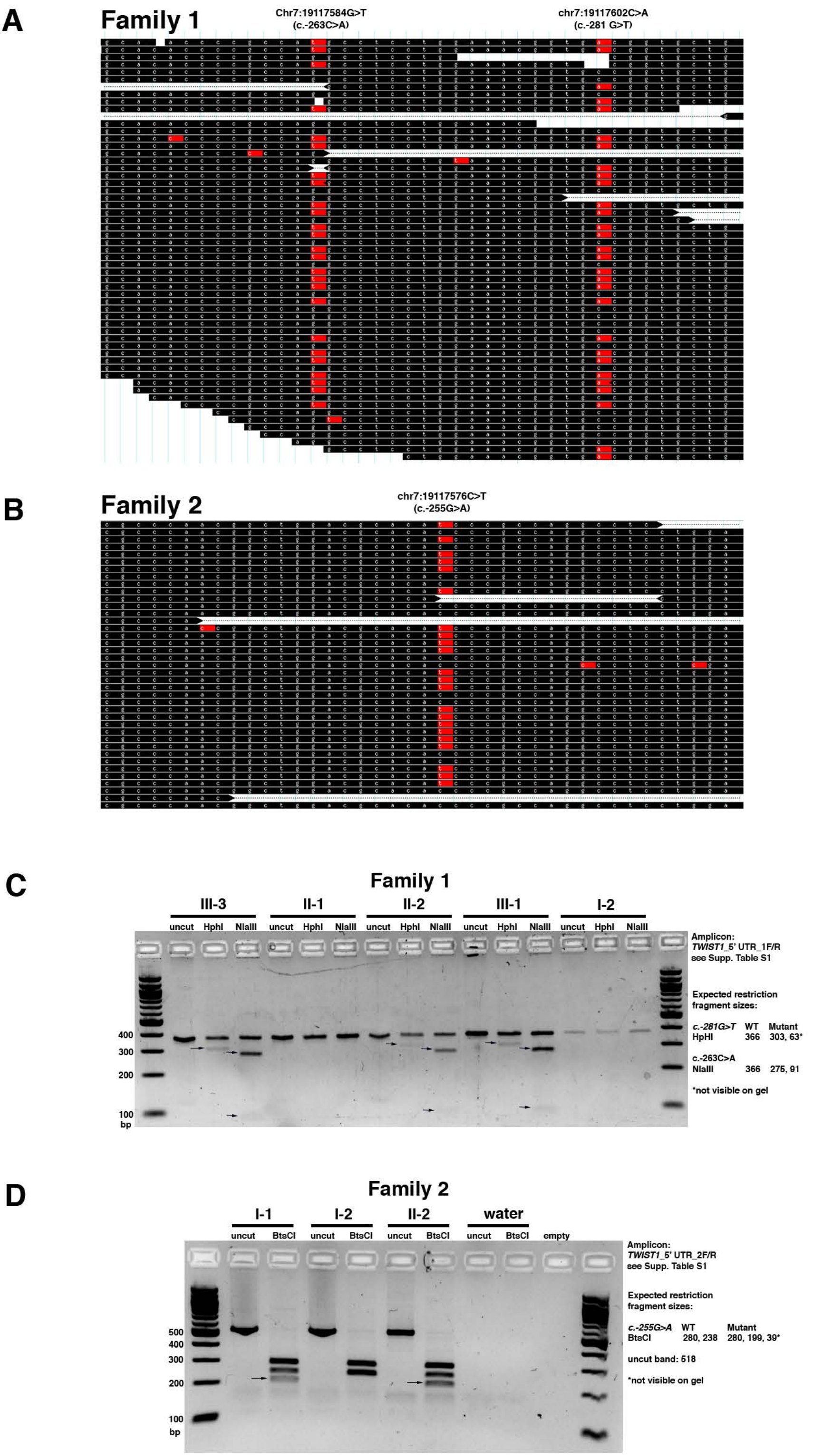

A, B, Gbrowse visualisation of variants detected in Families 1 and 2, respectively. Note that the variants in Family 1 are in *cis*. C, D, confirmation of variants by restriction digest analysis.

Supplementary Figure S2. Alignment of vertebrate *Twist1* 5′ UTR sequences

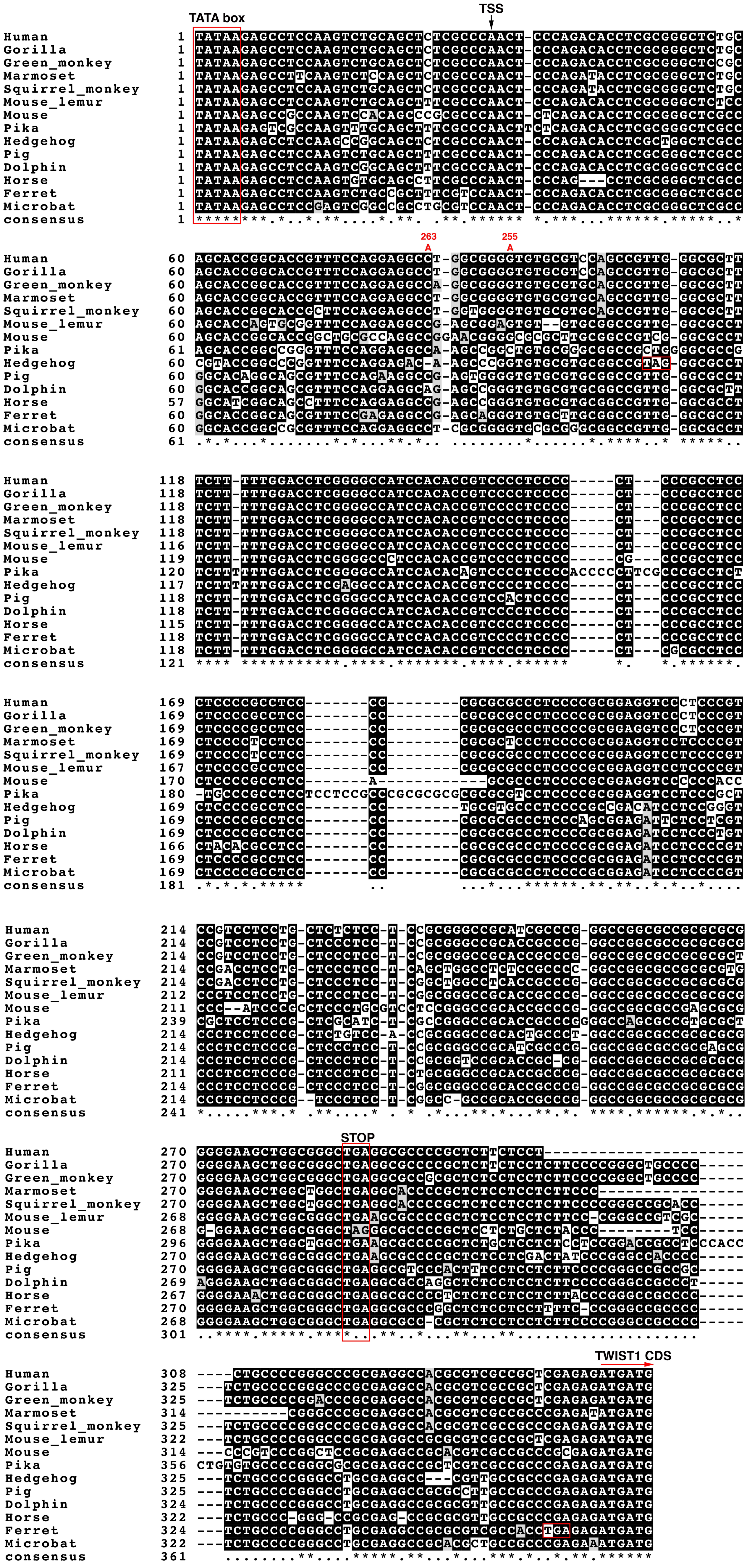

Note - alignment taken from TATA box to first 2 codons of *Twist1*. The highly conserved upstream STOP codon is boxed. The only other upstream STOP codons are in Hedgehog and Microbat (red boxes). The positions of the uAUG-creating variants identified in this study are indicated above the alignment. TSS, transcription initiation site (from Human: Wang et al., (1997) Cloning of the human *twist* gene: Its expression is retained in adult mesodermally-derived tissues. Gene 187:83-92).

Supplementary Figure S3. *TWIST1* 5' UTR - SNVs and uATGs

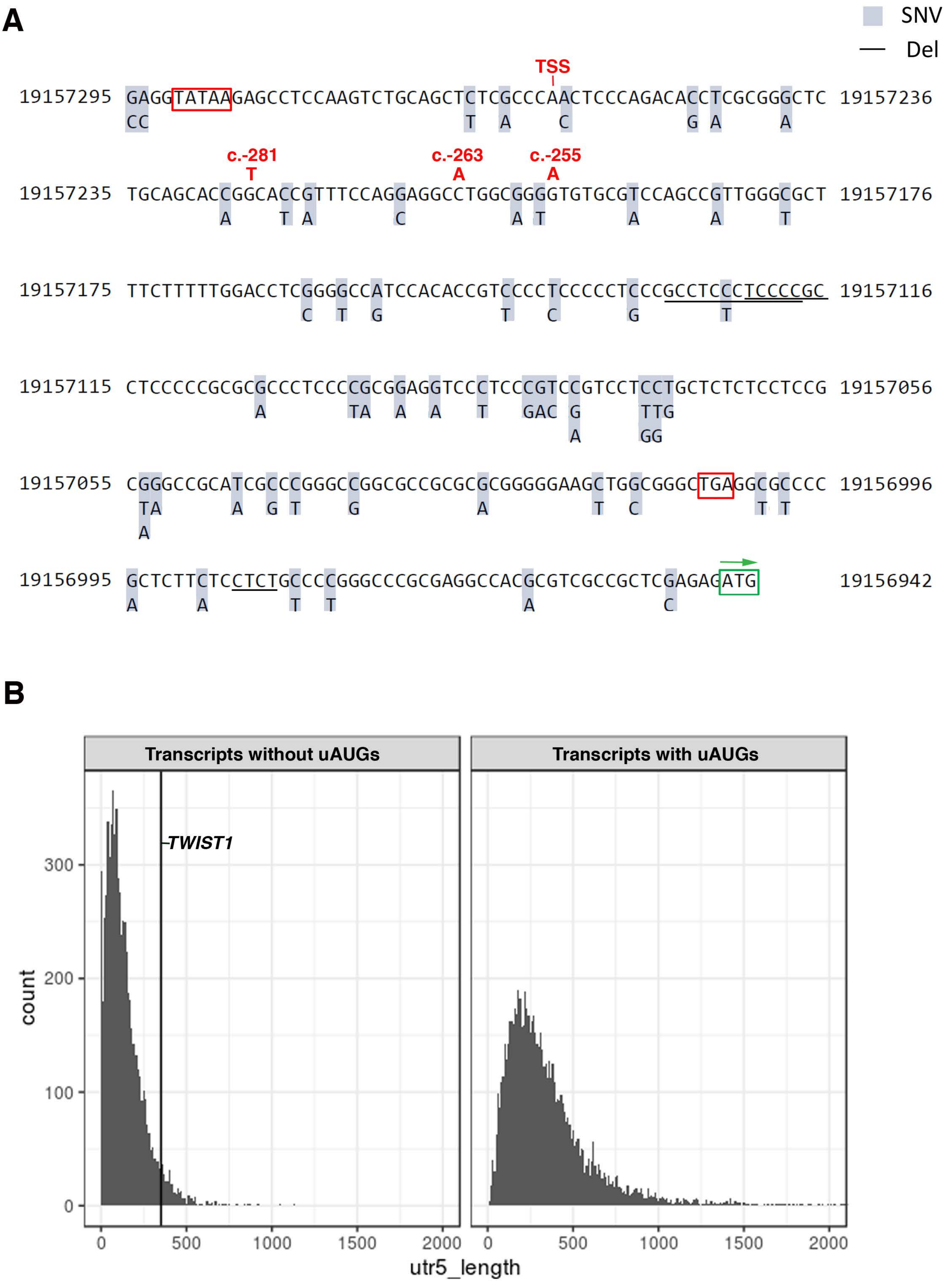

A, DNA sequence upstream of the *TWIST1* sAUG (green box). Positions that are polymorphic (gnomAD database) are highlighted in light blue with single nucleotide variants (SNVs) shown in the second row of sequence at each location. Where there are 3 possible variants there is a third row. Polymorphic deletions are underlined. The highly conserved upstream stop codon (TGA) is boxed in red. TSS, transcription start site. There are no natural uAUGs in the *TWIST1* leader sequence. B, Histogram of 5' UTR lengths of all high-quality transcripts in Gencode, split up by whether they have an uAUG (right hand plot) or not (left hand plot). *TWIST1* does not contain an uAUG, and is indicated by the vertical line; this indicates that *TWIST1* is amongst a smallergroup of genes with longer UTRs that do not contain uAUGs.

Supplementary Table S1. Resequencing coverage statistics

chr7:19,115,468-19,117,672 (GRCh38)	**Median**	**Minimum**	**Maximum**
Average region coverage	133x	19x	484x
Percentage of region covered at 10x or higher	98%	73%	100%
Percentage of region covered at 30x or higher	83%	20%	100%

Supplementary Table S2. Primers and amplification conditions

**Primers used for validation PCR, dideoxy sequencing and psi-check2 reporter cloning**[a]			
Amplicon	Forward	Reverse	Amplicon size (bp)
*TWIST1*_5' UTR_1	GAGGTATAAGAGCCTCCAAGTC	CACGTCCTGCATCATCTCTC	366
*TWIST1*_5' UTR_2	GGACTGGAAAGCGGAAACT	CTCTTCCTCGCTGTTGCTC	518
*TWIST1*_5' UTR_reporter	gttcgggctAGCCTCCAAGTCTGCAGC	caggcgctagCATCTCTCGAGCGGCGAC	344

**Primers used for site-directed mutagenesis**[b]			
Amplicon	Forward	Reverse	PCR Template [c]
Dual (c.-281 & c.-263)	CAGGAGGCaTGGCGGGGTGTGCGTCCA	GAAACGGTGaCGGTGCTGCAGAGCCCGC	psiCheck2_TWIST1_WT
c.-281	TGCAGCACCGtCACCGTTTCC	GAGCCCGCGAGGTGTCTG	
c.-263	TCCAGGAGGCaTGGCGGGGTG	AACGGTGCCGGTGCTGCAG	
c.-255	GCCTGGCGGGaTGTGCGTCCA	CTCCTGGAAACGGTGCCGGTG	
c.-263 + c.-252STOP	TGGCGGGGTGaGCGTCCAGCC	TGCCTCCTGGAAACGGTGCC	psiCheck2_TWIST1_c.-263
c.-255 + c.-246STOP	GATGTGCGTCtAGCCGTTGGG	CCGCCAGGCCTCCTGGAA	psiCheck2_TWIST1_c.-255
c.-255 + c.-75STOP	GCGCGCGGGGtAAGCTGGCGG	GGCGCCGGCCCGGGCGAT	psiCheck2_TWIST1_c.-255

**Primers used for RT-qPCR**[d]			
Amplicon	Forward	Reverse	Amplicon size (bp)
Renilla luciferase	TCCATGCTGAGAGTGTCGTG	CAAGCACCATTTTCTCGCCC	108
Firefly luciferase	TCTGGCGACATTGCCTACTG	CGGCGTCGAAAATGTTAGGG	145

Note – [a]PCR amplification of the *TWIST1* 5' UTR region was performed in a reaction volume of 25 µl, using 5X KAPA HiFi Buffer with 1.5 µl MgCl2 (25mM), 0.5 µl DMSO, 0.5 µl 10mM dNTP mix, 0.5 U of KAPA HiFi DNA polymerase, 0.3 µM primers and 10 ng DNA. Cycling conditions employed a touchdown approach as follows: 3 min initial denaturation step at 95°C, then 20 cycles of 95°C for 20 s, 64°C (decreased by 0.5°C every cycle to 54°C) for 20 s, 72°C for 60 s, then 20 cycles of 95°C for 20 s, 54°C for 20 s, 72°C for 60 s, and a final extension of 72°C for 2 min. [b]Site-directed mutagenesis was performed using the Q5 Site-Directed Mutagenesis kit (NEB #E0552S) following the manufacturer's instructions. Lowercase letters within the primer sequences indicate the mutated bases. [c]PCR templates used in the site-directed mutagenesis assay are listed accordingly. [d]Total RNA was obtained using the RNeasy Mini kit (QIAGEN 74104), following the manufacturer's instructions. cDNA synthesis was performed using the SuperScript™ IV First-Strand Synthesis System (Invitrogen™ 18091050) and oligo-dT primers. qPCR reactions were performed with KAPA SYBR® FAST (KK4610) in a 20 µl volume on a Roche LightCycler®480 II qPCR machine.
